# Effect of Remote Ischemic Preconditioning on Phosphorylated Protein Signaling in Children Undergoing Tetralogy of Fallot Repair: A Randomized Controlled Trial

**DOI:** 10.1161/JAHA.113.000095

**Published:** 2013-06-21

**Authors:** Salvatore Pepe, Norman Y. Liaw, Michele Hepponstall, Freya L. Sheeran, Matthew S. Yong, Yves d'Udekem, Michael M. Cheung, Igor E. Konstantinov

**Affiliations:** 1 Heart Research Group, Murdoch Childrens Research Institute, University of Melbourne, Melbourne, Australia (S.P., N.Y.L., F.L.S., Y.U., M.M.C., I.E.K.); 2 Department of Paediatrics, University of Melbourne, Melbourne, Australia (S.P., N.Y.L., M.H., F.L.S., M.S.Y., Y.U., M.M.C., I.E.K.); 3 Department of Cardiology, Royal Children's Hospital, Melbourne, Australia (M.M.C.); 4 Department of Cardiac Surgery, Royal Children's Hospital, Melbourne, Australia (M.S.Y., Y.U., I.E.K.)

**Keywords:** cyanosis, heart, mitochondria, pediatric surgery, remote ischemic preconditioning, tetralogy of Fallot

## Abstract

**Background:**

Our previous randomized controlled trial demonstrated cardiorespiratory protection by remote ischemic preconditioning (RIPC) in children before cardiac surgery. However, the impact of RIPC on myocardial prosurvival intracellular signaling remains unknown in cyanosis. RIPC may augment phosphorylated protein signaling in myocardium and circulating leukocytes during tetralogy of Fallot (ToF) repair.

**Methods and Results:**

Children (n=40) undergoing ToF repair were double‐blind randomized to RIPC (n=11 boys, 9 girls) or control (sham RIPC: n=9 boys, 11 girls). Blood samples were taken before, immediately after, and 24 hours after cardiopulmonary bypass. Resected right ventricular outflow tract muscle and leukocytes were processed for protein expression and mitochondrial respiration. There was no difference in age (7.1±3.4 versus 7.1±3.4 months), weight (7.7±1.8 versus 7.5±1.9 kg), or bypass or aortic cross‐clamp times between the groups (control versus RIPC, mean±SD). No differences were seen between the groups for an increase in the ratio of phosphorylated to total protein for protein kinase B, p38 mitogen activated protein kinase, signal transducer and activator of transcription 3, glycogen synthase kinase 3β, heat shock protein 27, Connexin43, or markers associated with promotion of necrosis (serum cardiac troponin I), apoptosis (Bax, Bcl‐2), and autophagy (Parkin, Beclin‐1, LC3B). A high proportion of total proteins were in phosphorylated form in control and RIPC myocardium. In leukocytes, mitochondrial respiration and assessed protein levels did not differ between groups.

**Conclusions:**

In patients with cyanotic heart disease, a high proportion of proteins are in phosphorylated form. RIPC does not further enhance phosphorylated protein signaling in myocardium or circulating leukocytes in children undergoing ToF repair.

**Clinical Trial Registration:**

URL: (http://www.anzctr.org.au/trial_view.aspx?id=335613. Unique identifier: Australian New Zealand Clinical Trials Registry number ACTRN12610000496011.

## Introduction

The cardioprotective phenomenon of ischemic preconditioning (IPC) involves the induction of brief, repeated episodes of myocardial ischemia and reperfusion to reduce the size of myocardial infarction after sustained ischemia–reperfusion (IR). A more practical, noninvasive, and clinically relevant model for protection is remote IPC (RIPC), whereby acute intermittent ischemia induced at a distant site (ie, interruption of blood flow to a limb) protects against IR injury in remote organs, including the heart.^1–3^

RIPC involves regulatory phosphorylation of a number of key intracellular proteins that propagate signaling for prosurvival metabolic control in the heart, such as Akt (protein kinase B), p38 mitogen activated protein kinase (p38MAPK), signal transducer and activator of transcription 3 (STAT3) protein, glycogen synthase kinase 3β (GSK3β), heat shock protein 27 (HSP27), and Connexin43.^4–10^ The regulation of proapoptotic and antiapoptotic proteins appears to occur through the interplay of Bax and Bcl‐2, with an increased ratio of Bax to Bcl‐2 being indicative of the initiation and progression of cell death.^11^ Autophagy is also an integral process of cell survival involving the selective degradation of long‐lived cellular components, with Beclin1, Parkin, and Light chain 3B (LC3B) contributing to key steps from the formation of the autophagosome to the final autolysosome.^12^ Although autophagy is a conserved and restricted process under normal conditions, hypoxia, nutrient deprivation, and mitochondrial dysfunction significantly augment autophagy to remove damaged components and make available free amino acids and fatty acids for energy production.

Protection against IR injury is of utmost importance in children undergoing heart surgery, as prolonged periods of cardioplegic arrest and cardiopulmonary bypass (CPB) are often required. We have previously demonstrated that RIPC provided clinically relevant protection in children undergoing heart surgery with CPB for congenital heart defects.^2^ Studies of RIPC in a porcine model of IR demonstrated improvement in lung compliance, reduction in the size of myocardial infarction, and amelioration of diastolic dysfunction.^13–14^ However, the efficacy of RIPC in the clinical setting is controversial due to reported absence of benefits after cardiac surgery.^15^ The efficacy of RIPC in children with cyanotic heart disease remains unknown. Are these children already “preconditioned” due to chronic hypoxia since birth? Thus, we tested the effect of RIPC in a homogeneous group of patients undergoing repair for tetralogy of Fallot (ToF) and measured the phosphorylation state of key signaling proteins associated with IR in these chronically hypoxic children.

## Methods

### Patient Recruitment and Allocation

This study was approved by the Royal Children's Hospital Human Ethics Committee and strictly conforms to the National Health and Medical Research Council of Australia Statement on Ethical Conduct in Human Research (2009). Signed consent was obtained from the patient's guardian before enrollment in the study. This prospective double‐blind randomized trial was registered (June 16, 2010) with the Australia and New Zealand Clinical Trials Registry (ACTRN12610000496011).

Patients diagnosed with ToF (n=40), aged 1 month or older, and undergoing elective open heart surgery with standard blood cardioplegia and CPB were included in this study. Those presenting with chromosomal defects, associated congenital lung malformations, or hematological disorders were excluded. Patients were randomized to 1 of 2 treatment groups: control (sham treatment: placement of noninflated cuff on limb) or single‐limb RIPC.

### RIPC and Surgical Procedures

Immediately after the induction of standard anesthesia in the operating room, RIPC was induced by four 5‐minute cycles of single‐leg ischemia interspersed with equivalent periods of reperfusion using a blood pressure cuff (Welch Allen) that was inflated to 30 mmHg above the patient's systolic pressure. Blood flow interruption and restoration were monitored by pulse‐oximetry. Control patients received only noninflated pressure cuff placement. Surgery was performed using standard anesthetic agents, cardioplegic heart arrest, and CPB. Resected right ventricular outflow tract (RVOT) muscle was sampled during ToF repair.

### Leukocyte Preparation

At baseline, immediately post‐CPB (PCPB), and 24 hours PCPB, whole blood was obtained from each patient from an arterial line and collected in standard EDTA tubes. Samples were transferred to the laboratory within 30 minutes and underwent differential centrifugation to isolate leukocytes with Histopaque‐1077 (Sigma‐Aldrich) according to the manufacturer's instructions. Isolated leukocytes were counted with a Countess Automated Cell Counter (Invitrogen), and the percentage of total viable cells was assessed on the basis of permeability to trypan blue.

### Mitochondrial Oxygen Respiration

Isolated leukocytes were permeabilized with digitonin (100 μg/mL) in sucrose buffer (sucrose, HEPES 10 mmol/L, pH 7.4, EDTA 1 mmol/L, 5 minutes) in the presence of protease inhibitor mixture (Roche), 4‐(2‐aminoethyl)‐benzenesulfonyl‐fluoride hydrochloride (1 mmol/L; Roche), and di‐isopropyl fluorophosphate (2 mmol/L; Fluka). Cells were washed twice in sucrose buffer via centrifugation (1000*g*, 4°C, 5 minutes) to remove digitonin and then resuspended in sucrose buffer. States III and IV substrate‐coupled mitochondrial O_2_ consumption was measured via use of a miniature Clarke‐type O_2_ electrode in a closed‐volume 30 μL glass chamber (37°C) in glutamate/malate buffer (2.5/0.5 mmol/L), driven by ADP (0.5 mmol/L). Maximal O_2_ consumption rate was determined with the proton gradient uncoupler carbonyl‐cyanide‐4‐(trifluoromethoxy)phenylhydrazone (FCCP, 2 μmol/L). Nonmitochondrial sources of O_2_ consumption were assessed after the addition of sodium cyanide (5 mmol/L, complex IV inhibitor) and subtracted from total O_2_ consumption. Stoichiometric estimates of ADP:O consumption were determined as an index of mitochondrial oxygen utilization efficiency in the consumption of ADP during ATP formation.

### Protein Extraction and Concentration Determination

One milliliter of modified radioimmunoprecipitation assay (RIPA) buffer was added to every 0.1 g of RVOT heart tissue or total leukocyte isolation with phosphatase and protease inhibitors (Sigma‐Aldrich). Tissue was homogenized with a TissueRuptor (Qiagen) and centrifuged (2000*g*, 10 minutes, 4°C). Supernatants were measured for total protein via bicinchoninic assay (Sigma‐Aldrich).

### Immunoblotting

Protein expression was determined by standard western blotting conditions of RVOT and leukocyte extracts. Lanes (4 to 10 μg protein) were separated on a 10% or 15% SDS‐PAGE gel and electrotransferred to a PVDF membrane at 100 V for 1.5 hours. Membranes were enclosed in a blot holder and blocked with TBS‐Tween (TBST)+0.5% skim milk (TBST/skim) using the Snap i.d. System (Merck‐Millipore). Primary antibody incubation of the membrane with phosphorylated (p)‐Akt (1:2000), Akt (1:1000), p‐p38MAPK (1:1000), p38MAPK (1:1000), p‐GSK3β (1:1000), GSK3β (1:1000), p‐STAT3 (1:1000), STAT3 (1:1000), p‐HSP27 (1:1000), HSP27 (1:1000), p‐Connexin43 (1:1000), Connexin43 (1:1000), Bcl‐2 (1:1000), Bax (1:1000), Parkin (1:1000), LC3B (1:1000), and Beclin1 (1:1000) occurred at room temperature on a blood‐tube roller for 1 hour. All primary antibodies were purchased from Cell Signaling Technologies. Detected target proteins were normalized to protein expression of GAPDH or β‐tubulin.

Membranes were transferred to a blot holder, washed 3 times with TBST under vacuum, incubated with either goat antirabbit or goat antimouse secondary antibodies (HRP conjugated, 1:2000 dilution; Bio‐Rad) for 10 minutes and, subsequently, washed an additional 3 times. Protein bands were detected via enhanced chemiluminescence (Perkin Elmer) with membranes exposed to film (Amersham). Film was scanned at a minimum resolution of 600 dpi, and densitometries were analyzed with use of ImageJ (National Institutes of Health).

### Cardiac Troponin I

Cardiac‐specific troponin I (cTnI) released after cardiac myocyte injury was assayed in blood serum samples collected at baseline and up to 24 hours PCPB using the VITROS 5600 Integrated System and VITROS Immunodiagnostic Troponin I ES Reagent and Calibrator packs (Ortho Clinical Diagnostics Inc).

### Statistical Analysis

The Fisher exact test was used to determine whether gender distribution was similar between allocated treatment groups. The Shapiro–Wilk test for normality was used to determine whether our data had a normal distribution. For normally distributed data, linear mixed models were performed with Bonferroni correction for post‐hoc multiple contrasts. Data are presented as mean±SD. Data that were not normally distributed were presented as median and interquartile range and contrasted for group differences by the Friedman test and Wilcoxon signed‐rank test performed with Bonferroni adjustment for multiple measures. Data were analyzed using the SPSS statistical software package, version 19 (IBM), and considered significant where *P*<0.05.

## Results

### Patient Anthropometric Data and Perioperative Conditions

Due to a predominantly uniform experimental cohort, particularly with regard to age, gender, body weight, and operative conditions (Table****1), no significant differences were observed between treatment groups. Patients in both groups received comparable levels of morphine, fentanyl, isoflurane, pancuronium, vecuronium, methyl prednisolone, tranexamic acid, cephazolin, midazolam, dobutamine, heparin, blood cardioplegia, and protamine as a routine part of the preparatory anesthetic and perioperative regimens.

**Table 1.** Patients' Perioperative Characteristics

	Control (n=20)	RIPC (n=20)	*P* Value
Male, female (n, n)	9, 11	11, 9	0.75
Age, mo	7.4±3.3 (3.3 to 14.2)	7.6±3.2 (3.3 to 14.2)	0.89
Weight, kg	7.7±1.8 (5.0 to 11.6)	7.5±1.9 (5.0 to 11.6)	0.71
Intraoperative parameters			
Minimum core body temperature, °C	31.6±2.1 (26.1 to 35)	33.1±1.8 (31.6 to 36.3)	0.07
Cardiopulmonary bypass time, min	104±7 (80 to 158)	94±7 (55 to 186)	0.98
Total aortic cross‐clamp time, min	83±22 (56 to 131)	75±20 (43 to 108)	0.36
Postoperative parameters			
Ventilation time, h	21 (IQR 14 to 38.5)	21 (IQR 19 to 23.8)	0.86
ICU stay, h	45.3 (IQR 26.5 to 57)	29.7 (IQR 25.3 to 47.7)	0.50
Hospital stay, d	12 (IQR 7 to 17.5)	7 (IQR 5.5 to 12.5)	0.09
Mean arterial pressure, mm Hg			
3 h	59.8±7.5 (45 to 72)	60.5±7.3 (46 to 74)	0.78
6 h	55.8±8.5 (40 to 71)	59.2±7.5 (47 to 80)	0.24
12 h	61±8.5 (41 to 74)	58.7±7.8 (46 to 75)	0.43
24 h	62.5±10.1 (44 to 81)	61.9±10.6 (42 to 86)	0.76
Right atrial pressure, mm Hg			
3 h	10 (IQR 9 to 14)	11 (IQR 10 to 13)	0.97
6 h	11 (IQR 10 to 15)	11 (IQR 9 to 13)	0.86
12 h	11 (IQR 9 to 13)	13 (IQR 9 to 14)	0.29
24 h	11 (IQR 9 to 12)	12 (IQR 10 to 13)	0.12
Alveolar–arterial gradient			
3 h	42.2 (IQR 22.6 to 66.6)	46.5 (IQR 37.4 to 122.9)	0.06
6 h	45.3 (IQR 26.6 to 81)	51.4 (IQR 26.9 to 94.2)	0.43
12 h	43.3 (IQR 26.8 to 67.5)	47.5 (IQR 24.8 to 74.5)	0.68
24 h	37.6 (IQR 23 to 76.5)	47.8 (IQR 33 to 93.7)	0.14
Wernovsky inotropic score			
3 h	2.25 (IQR 0 to 5)	1.8±2 (0 to 5)	0.53
6 h	2.25 (IQR 0 to 5)	1.8±2.2 (0 to 7.5)	0.55
12 h	2.25 (IQR 0 to 5)	2.2±2.7 (0 to 8)	0.57
24 h	2.25 (IQR 0 to 4.5)	1.9±2.7 (0 to 8)	0.89
Vasoactive inotropic score			
3 h	1.5 (IQR 0 to 5)	4 (IQR 0.3 to 5)	0.36
6 h	3 (IQR 0 to 5)	2.75 (IQR 0.5 to 5.75)	0.67
12 h	5 (IQR 0.75 to 11)	5 (IQR 2.5 to 5.75)	0.67
24 h	4 (IQR 0 to 5)	3.5 (IQR 0 to 5)	0.80
Lactate, mg/dL			
3 h	1.4±0.35 (1 to 2.1)	1.4±0.41 (0.9 to 2.4)	0.87
6 h	1.5±0.4 (0.95 to 2.7)	1.3±0.31 (0.7 to 1.8)	0.14
12 h	1.5±0.39 (0.8 to 2.1)	1.3±0.29 (0.81 to 1.82)	0.05
24 h	1.4±0.36 (0.72 to 2.5)	1.5±0.77 (0.79 to 4.01)	0.41
Creatinine, mg/dL			
3 h	30.1±7.6 (23 to 53)	31.2±4.3 (23 to 39)	0.14
12 h	42.3±10.4 (25 to 63)	39.8±6.9 (26 to 49)	0.42
Urea, mg/dL			
3 h	4.1±1.2 (1.1 to 7.3)	4.2±1.4 (1.1 to 7.3)	0.25
12 h	7.1±2.1 (2.2 to 12)†	7.2±2.7 (2.2 to 12)†	0.88
Cardiac troponin I, μg/L			
Baseline	0.025±0.03 (0.012 to 0.12)	0.019±0.02 (0.012 to 0.12)	0.67
0 h	6.8±3.7 (2.3 to 13.7)*	7.4±4.9 (1.3 to 19.7)*	0.58
6 h	11.2±5.4 (4.9 to 19)*	10.9±6.5 (4.7 to 25.4)*	0.94
24 h	3.6±1.6 (1.3 to 6.7)*‡	4.2±2.3 (1.5 to 9.2)*‡	0.49

Normally distributed data are presented as mean±SD (range) and nonparametric data as median (IQR [interquartile range]). For repeated measures: *P<0.05 vs baseline, ^†^*P*<0.05 vs 3 hours, ^‡^*P*<0.05 vs 6 hours.

Wernovsky inotropic scoring^16^ was used to assess the patient hemodynamic cardiac index, systemic and pulmonary vascular resistances, intraoperative and postoperative fluid balance, and perioperative course. Vasoactive‐inotropic score^17^ assessments were performed to determine total cardiovascular support from vasoactive drugs in the first 24 hours after CPB. No differences were evident between groups. No intraoperative or postoperative deaths occurred. There were no adverse events after the RIPC induction protocol.

### Mitochondrial Respiration

Despite the expected slight decline in mitochondrial oxygen consumption after CPB (PCPB and 24 hours), Table 2 shows relatively well‐coupled mitochondrial respiration in digitonin‐permeabilized leukocytes from both control and RIPC groups at all time points. No differences between the treatment groups were detected for basal or maximal oxygen consumption rates.

**Table 2.** Complex I Substrate‐Dependent (glutamate 5 mmol/L+Malate 2.5 mmol/L) Mitochondrial Oxygen Consumption Rate (nmol O_2_/min per 10^7^ leukocytes) in Digitonin‐Permeabilized Leukocytes Sampled at Baseline, Immediate PCPB, and 24 Hours PCPB

	Control			RIPC		
	Baseline	PCPB	24 h	Baseline	PCPB	24 h
State III	4.74±0.38	3.89±0.26*	3.84±0.29*	4.77±0.3	3.96±0.4*	4.0±0.3*
State IV	2.44±0.23	2.67±0.27*	2.98±0.31*†	2.4±0.2	2.56±0.26*	2.84±0.3*†
FCCP	4.96±0.38	4.84±0.32*	4.55±0.32*†	4.8±0.34	4.76±0.33	4.6±0.3*†
ADP:O	1.8±0.18	1.6±0.24*	1.46±0.18*†	1.88±0.22	1.7±0.2*	1.53±0.2*†

Values are mean±SD. State III respiration is stimulated by 0.5 mmol/L ADP; state IV respiration is the post‐ADP consumption rate. Maximal respiration is stimulated by the uncoupler FCCP (2 mmol/L). ADP:O is the oxygen utilization efficiency per unit of ADP consumed to form ATP. For each measure, there was no significant difference between control and RIPC. For Bonferroni‐adjusted repeated measures: *P<0.01 vs baseline, ^†^*P*<0.01 vs PCPB. PCPB indicates postcardiopulmonary bypass; RIPC, remote ischemic preconditioning; FCCP, carbonyl‐cyanide‐4‐(trifluoromethoxy)phenylhydrazone.

### Intracellular Prosurvival Signaling Protein Expression

**Figure 1.**
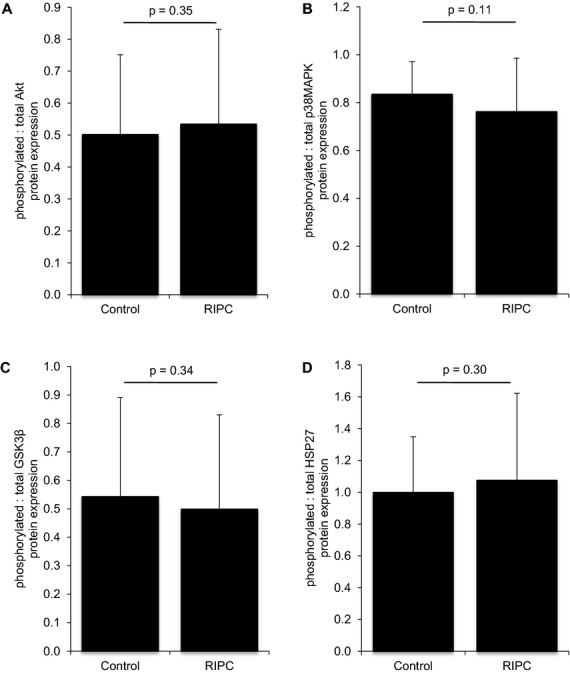
RVOT tissue protein expression of prosurvival signaling pathway kinases obtained during CPB from patients subjected to Control (Sham) or RIPC treatments. Western blots of protein bands were densitometrically detected using antibodies targeting phosphorylated and total Akt (A), p38MAPK (B), GSK3β (C), and HSP27 (D). Data (relative densitometric units) were normalized to loading controls detected by antibodies to either GAPDH or β‐tubulin and are presented as the ratio of phosphorylated to total protein expression (mean±SD). RVOT indicates right ventricular outflow tract; CPB, cardiopulmonary bypass; RIPC, remote ischemic preconditioning; p38MAPK, p38 mitogen activated protein kinase; GSK3β, glycogen synthase kinase 3β; HSP27, heat shock protein 27.

**Figure 2.**
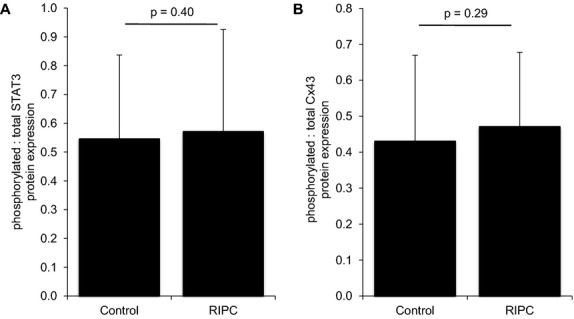
RVOT tissue protein expression of STAT3 (A) and Connexin43 (B), as described in the Methods section. Data (relative densitometric units) are normalized to loading controls and presented as the ratio of phosphorylated to total protein expression (mean±SD). RVOT indicates right ventricular outflow tract; STAT3, signal transducer and activator of transcription 3; RIPC, remote ischemic preconditioning.

We performed Western blotting to determine protein expression of prosurvival signaling intermediates from resected RVOT myocardium during repair of ToF. No significant difference was apparent between control and RIPC groups in the ratio of phosphorylated to total (p:t) proteins specifically probed for Akt (Figure 1A), p38MAPK (Figure 1B), GSK3β (Figure 1C), HSP27 (Figure 1D), STAT3 (Figure 2A), and Connexin43 (Figure 2B).

In contrast to RVOT tissue, in leukocytes isolated from blood samples measured immediately or 24 hours PCPB, phosphorylated fractions of Akt, p38MAPK, GSK3β, STAT3, Connexin43, and HSP27 were each evident only as extremely faint bands or not at all, even when protein loading in lanes was increased as high as 20 μg. However, detection of bands with abundant density and specificity for total Akt, p38MAPK, GSK3β, STAT3, Connexin43, and HSP27 was evident in the same protein aliquots, in as little as 4 μg/loading lane.

### Apoptotic, Autophagic, and Necrotic Markers of Myocardial Injury

**Figure 3.**
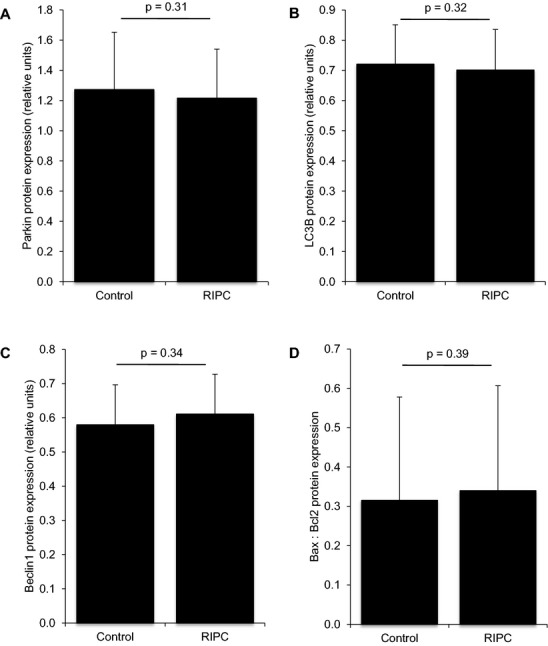
RVOT tissue protein expression of the autophagy proteins Parkin (A), light chain 3B (LC3B) (B), Beclin 1 (C), and the proapoptotic protein Bax and antiapoptotic Bcl‐2, expressed as the ratio Bax:Bcl‐2, (D). Data are presented as relative (densitometric) units or as a ratio (mean±SD). RVOT indicates right ventricular outflow tract; RIPC, remote ischemic preconditioning.

Immunoblotting detection demonstrated no significant differences between control and RIPC treatment groups for Parkin (Figure 3A), LC3B (Figure 3B), and Beclin1 (Figure 3C) or in the ratio of Bax to Bcl‐2 (Figure 3D). Table 1 summarizes the cTnI levels detected in serum from control and RIPC patients, with no significant differences evident. As expected due to surgical intervention, cTnI levels were significantly elevated compared with baseline immediately PCPB, rising further by 6 hours, but declining markedly by 24 hours PCPB (Table 1).

## Discussion

The previous study of RIPC in children undergoing CPB cardiac operations^2^ included a patient cohort with mixed congenital pathology (including ToF) and older age compared with the present study, thus differing primarily by developmental age, cardiac defects, and chronic cyanosis. The present study was undertaken to examine a more uniform patient cohort and to evaluate the extent of prosurvival kinase activity in response to RIPC. However, no impact of RIPC on the phosphorylation levels of prosurvival proteins in resected heart muscle from patients undergoing repair of ToF was apparent in the present randomized controlled trial. All kinases assayed were chosen based on their involvement in preconditioning reported in animal studies of IPC or RIPC, along with their activity on specific intracellular targets.^18^ Although the myocardial concentrations of these protein kinases were abundant, the phosphorylated form of each kinase was high relative to its total level. The high p:t ratio of kinase expression in controls (>50%, up to 80% to 100% of total) suggests that these “cardioprotective” signaling pathways were already activated by chronic hypoxia in cyanotic children.

RIPC stimulus in adult mice results in increased phosphorylation of both Akt and GSK3β in myocardium and invokes protection against subsequent reperfusion injury.^5^ Phosphorylation of GSK3β has been demonstrated to be a critical integration point of preconditioning pathways involving regulation of mitochondrial metabolism and permeability pore transition.^19^ p‐GSK3β has been shown to be colocalized in mitochondria with p‐Connexin43, which enables increased open probability of mitochondrial K_ATP_ channels and cardioprotection.^20^ Connexin43 is an integral membrane protein whose role in gap junction formation is essential in maintaining electrical and mechanical coupling between cardiomyocytes; however, phosphorylation of Connexin43 enables translocation to mitochondria to permit cardioprotection.^20–21^ In our study, p‐Connexin43 levels were high but similar in both groups. In leukocytes, no difference in mitochondrial respiration was detected between the groups.

Preconditioning limits the proapoptotic signaling of Bax, protects against cytochrome c release during IR injury, and preserves NADH‐linked ADP‐dependent mitochondrial respiration.^11^ An increased ratio of Bax to Bcl‐2 protein expression is indicative of an ongoing apoptotic process. However, no significant difference was evident between control and RIPC groups in Bcl‐2 or Bax expression. The autophagy markers Parkin, Beclin1, and LC3B, important to poststress cell survival,^12^ also did not differ between groups. Finally, in assessing the impact of RIPC on the propensity for cardiomyocyte necrosis, cTnI release was measured in serum, but levels did not differ between the groups in the 24‐hour PCPB period. However, it is important to note that the levels of cTnI, which have been shown to be released postoperatively from cardiac surgery ToF patients with comparable CPB times, are greater (≥10‐fold) than levels evident in the present study.^22–24^

### Study Limitations

The current study was restricted to excised RVOT tissue and leukocytes. The advantage of studying leukocytes is the capacity to sample time points before and after RIPC and CPB. However, despite high concentrations of total protein assayed in isolated leukocytes, phosphorylated proteins were at or below the threshold of detection and thus were not quantifiable as per the RVOT tissue studies. This is in contrast to a pilot study of RIPC in healthy adults performed by our group, whereby 4 μg of total protein derived from isolated leukocytes was sufficient to quantifiably detect alterations in p:t protein ratios (not shown). Although mitochondrial respiration appeared normal, it is unclear whether leukocytes from infants have developmental differences in RIPC‐dependant kinase signaling compared with those from adults, because the majority of RIPC studies have been performed in adults. Our previous work has demonstrated that RIPC invokes a rapid genomic response in circulating leukocytes that involves suppression of genes encoding proteins involved in cytokine synthesis, leukocyte chemotaxis, adhesion and migration, exocytosis, innate immunity signaling pathways, and apoptosis.^25^ A complex global response to the plasma proteome has been recently demonstrated in response to RIPC stimulus that involves differential shifts in the presence of 51 proteins affecting a multitude of processes, including the immune response, hemostasis, and inflammation.^26^ In particular, we have recently demonstrated in human neutrophils a specific reduction in kinin receptor expression,^27^ adhesion, and functional responses after RIPC.^28^

Another limitation relates to the absence of an acyanotic group of infants undergoing CPB surgery. The lack of difference in the abundance of phosphorylated signaling proteins between the RIPC and control groups may relate to existing adaptations to chronic hypoxia. Recent data from fetal chick studies indicate that hypoxia and associated ROS directly increase the myocardial activity of MAPK and related signaling proteins, especially in ventricular outflow tract tissues.^29^ A qualitative study of right atrial tissue from cyanotic and acyanotic children undergoing surgery for cardiac congenital disorders reported detection of phosphorylated forms of p38MAPK, HSP27, JUN kinase, and protein kinase Cε only in chronically hypoxic patients.^30^ Thus, any additional benefit of RIPC in ToF may have been abrogated due to preceding adaptive increases in phosphorylated signaling proteins induced by chronic hypoxia. Notably, this adaptation may be influenced by the markedly high levels of endogenous myocardial opioid peptides, which correlate inversely with the extent of arterial oxygen saturation, in children with ToF.^31^

Pharmacological preconditioning, including volatile anesthetic agents such as isoflurane routinely used during surgery, may have also contributed to the lack of RIPC effect.^15^ It has been recently proposed that the ineffective results of RIPC in a few small trials may specifically relate to the type of volatile anesthetic used, combinations of agents, and their perioperative timing.^32^ Although volatile anesthetics have been found to exert direct cardioprotective effects in animal studies and facilitate RIPC efficacy (ie, isoflurane), others (ie, propofol) may impede or counteract RIPC effects. However, coronary artery bypass graft surgery in middle‐aged and elderly adults is markedly different in multiple facets compared with that in infants undergoing cardiac surgery for ToF, especially as advanced age has been reported to diminish the efficacy of preconditioning.^33^ In our current study, both groups received isoflurane and the opiates morphine and fentanyl.

## Conclusions

In the setting of cardiac surgery for ToF, in this first randomized clinical trial of RIPC in cyanotic infants, we demonstrate that although RIPC is safe, inexpensive, and easy to deploy, it does not further augment expression of prosurvival intracellular kinase signaling. Whether this lack of additional response to RIPC is due to prior adaptation to chronic hypoxia or the early age‐related underdevelopment of receptor‐coupled protein kinase intracellular signaling pathways or to the use of drugs routine to surgery that may evoke pharmacological preconditioning requires direct molecular and functional study. The present study findings emphasize that chronic patient adaptations and developmental age are crucial considerations to directly control in the design of future randomized clinical trials of RIPC.
